# Magnetic alignment in free-ranging Indian Leopard (*Panthera pardus fusca*)

**DOI:** 10.1371/journal.pone.0266129

**Published:** 2022-07-08

**Authors:** Reuven Yosef, Swapnil Kumbhojkar, Bablu Gurjar, Jakub Z. Kosicki

**Affiliations:** 1 Ben Gurion University of the Negev-Eilat Campus, Eilat, Israel; 2 Jhalana Wildlife Research Foundation Gharkul Society, Pune, India; 3 Department of Avian Biology and Ecology, Adam Mickiewicz University, Poznań, Poland; Amity University, INDIA

## Abstract

The earth’s geomagnetic field (GMF) is known to influence the behaviour of a wide range of species, but remains one of the most enigmatic of animal senses. Animals are known to utilize the GMF for a wide range of survival capabilities such as navigation and orienteering, migration, territoriality, homing, etc. Despite a lot of study in this regard on vertebrates, little is known about the effects of GMF on felids. Hence, we analyzed the body alignment of the Indian Leopard during defecation, and walking along the trails in the Jhalana Reserve Forest in India. Using circular statistics, we found that the leopards aligned their bodies on the north-south axis during defecation (mean azimuth -176.4°), while no such preference was found when walking (mean azimuth 52.9°). Thus we prove that leopards are sensitive to the GMF during basic physiological activities and in this context show similar behaviour to other vertebrates studied to date.

## Introduction

In recent years it was discovered that animals align their bodies along the Earth’s geomagnetic alignment (GMF, [1, 2]), but remains one of the most enigmatic of animal senses [3]. These magnetoreception capabilities and mechanisms are attributed mostly to biogenic magnetite [4, 5], to magnetic polarity in birds [6], or is based upon blue-light dependent receptors (cryptochromes) located within eyes, proposes the coupling of magnetic sensing with vision (radical pair hypothesis, [7]), in which both magnetic inclination and polarity play an important role [8]. It was found that specialized photoreceptors in the retina of the animals facilitated GMF perception in many groups of invertebrates and vertebrates e.g., [9–11].

The advantages accorded to the animals can be manifold wherein the magnetic fields are perceived as visual or other sensory patterns. Hunting red foxes (*Vulpes vulpes*) appear to use magnetic alignment (MA) to estimate distance to their prey [8]; wolves (*Canis lupus*) use the magnetic tool as an independent space frame that helps to mark and remember important places and territorial boundaries (magnetic map sense; [12, 13]); provides universal navigational information, a magnetic vector used as a compass or to orientate itself based on the intensity or latitudinal inclination of the magnetic field [14, 15]; or for homing by displaced individuals (e.g., reintroduction programs [16]). It is also used by subterranean animals to dig tunnels in a species-specific direction [17]. But it was [18, 19] who initially discovered the phenomenon when plotting whale strandings in the British Isles and found that they occurred at sites with locally decreased magnetic fields.

To date, there is a preponderance for studies of GMF in primates with a stress on humans (see [20] for review), in domestic animals e.g., [21], or wildlife in captivity [22], but [20] concluded that studies of wild animals in the field are under-represented. This is especially true for studies concerning felids. Indeed, in a survey of the scientific literature, there were no studies of magnetoreception in any of the big cats. Thus in this study we address this issue and we described directional preferences of Indian Leopards (*Panthera pardus fusca*) in natural habitats.

The simplest response to GMF that can be studied with relative easiness is the spontaneous orientation of the body during defecating [5]. Based on this assumption we checked in which geographical direction was the leopards’s head oriented during defecation. The determining of direction of the panther’s body during this physiological process was possible based on the specific behavior preceding defecation i.e., expressive scratching of the ground with paws (see movie: https://www.facebook.com/varad.bansod/videos/1800706399987528) and the manner in which the scats exit the body during the process.

## Methods

### Study area

**Fig 1:**
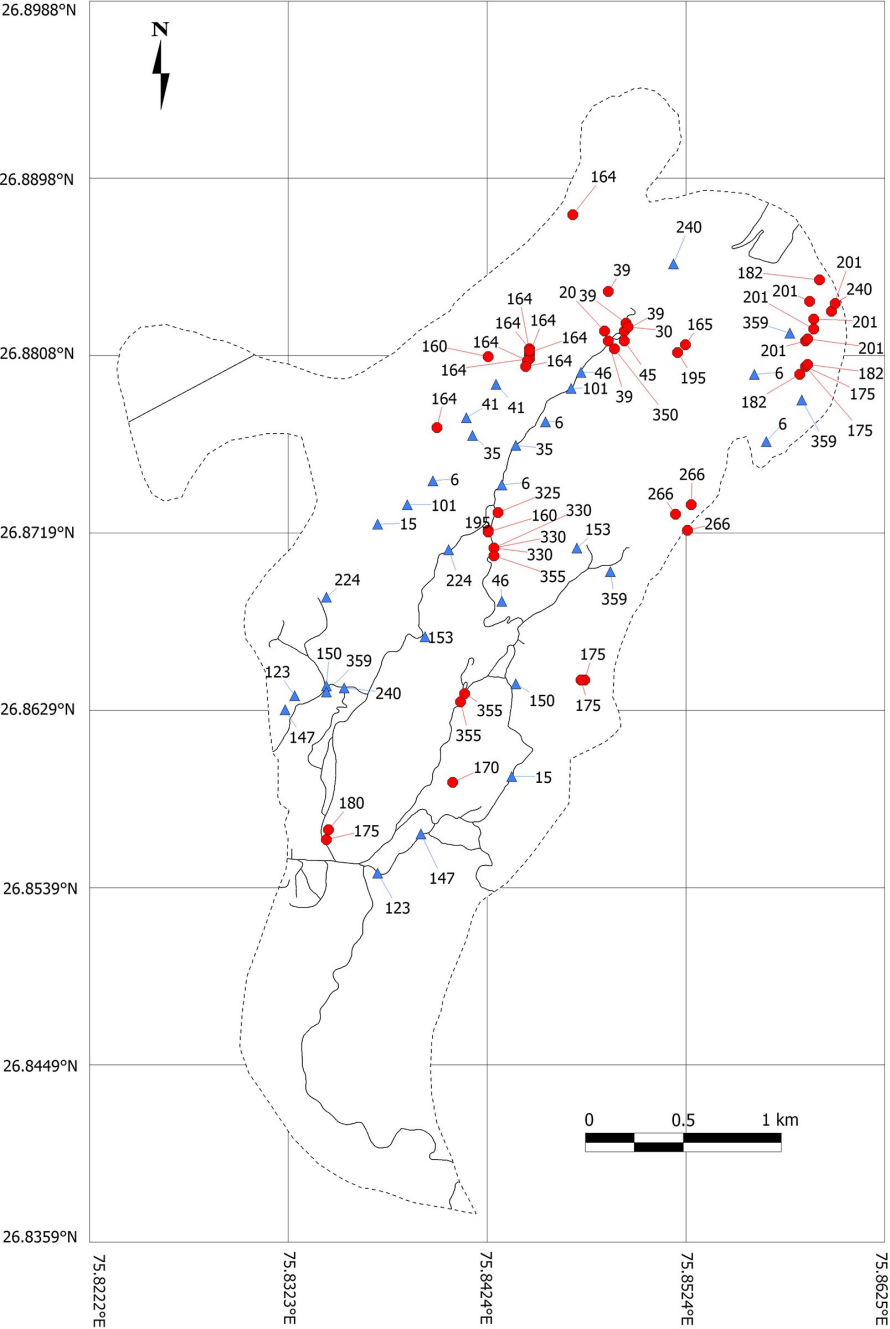
Map of the Jhalana Forest Reserve. Red circles denote the location of defecation, blue triangles leopards (*Panthera pardus fusca*) walking, numbers indicate azimuth of head orientation, and black lines represent roads or dirt tracks.

The Jhalana Reserve Forest (JRF; 26°51’N 75°49’E, 516 m AMSL, Fig 1) was initially the hunting grounds of the Maharaja of Jaipur, and although designated as a Reserved Forest in 1961 in accordance with the provisions of Rajasthan Forest Act 1953, it was declared a protected area in May 2017. JRF has a total area of 29 km^2^ and sustains a population of ca. 35 individuals of the Indian Leopard [23–25].

### Scat collection

**Fig 2:**
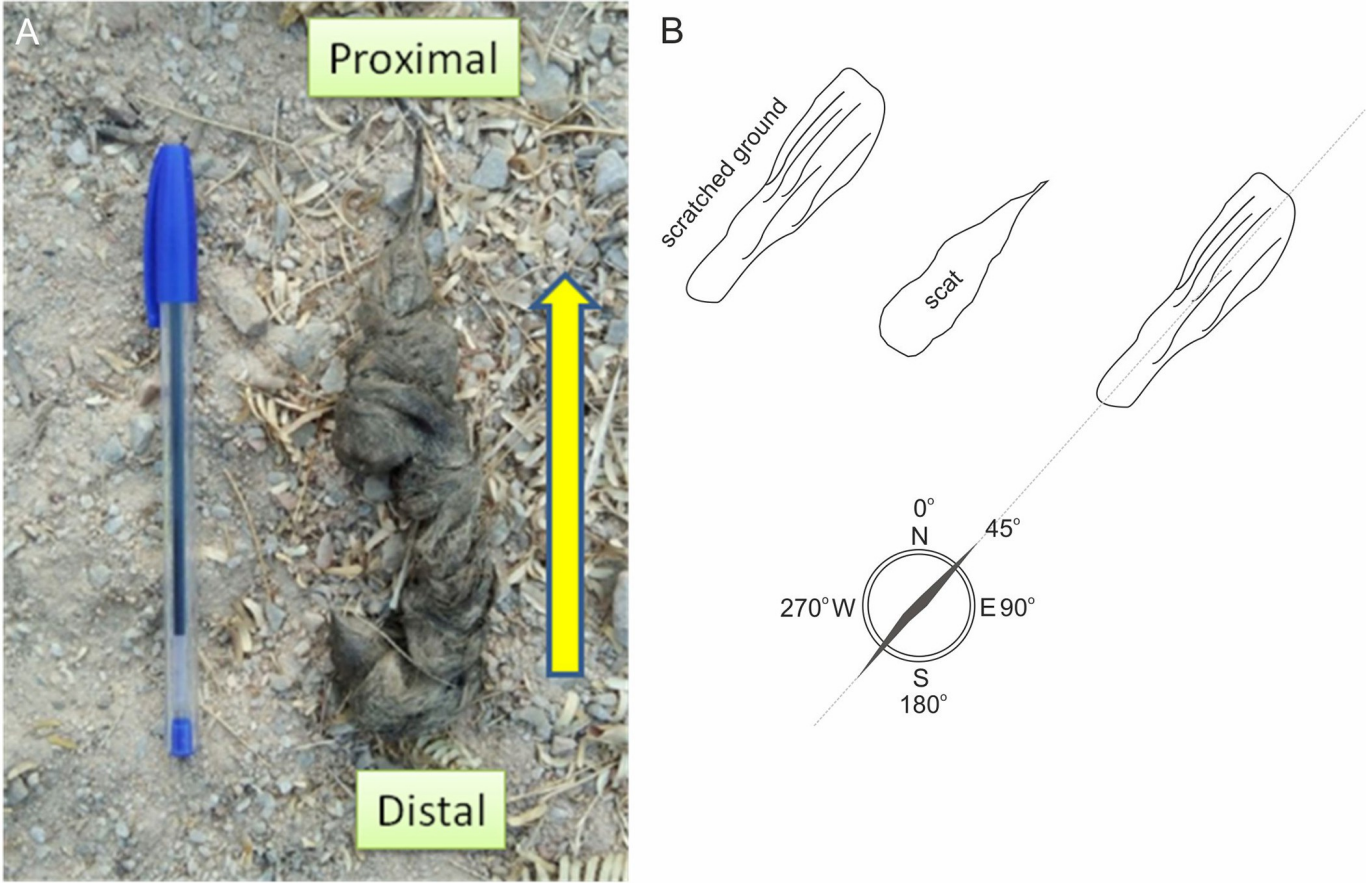
The field techniques used to identify the body alignment of the leopard (*Panthera pardus fusca*) by the elongated part of the scat (A), and a diagram illustrating body orientation during defecation (B). The yellow arrow depicts the direction of the head.

On eight different days (17, 20, 31 May, 3, 9, 13, 15, 19 June 2021), before the onset of the monsoon rains, we followed trails frequented by the leopards, which were determined on the basis of research conducted at present (Kumbhojkar et al., 2020a). All observations occurred between 0930 and 1230 hrs of the dates specified, and as restricted by the Rajasthan Forest Department for all tourist vehicles (ESM1). The trails are uniformly laid out such that there is open area on both sides for several tens of meters for viewing wildlife. These are also the areas where leopards can be observed at rest or their defecations. During defecation, the leopards scratch the ground with their hind legs (see https://www.facebook.com/varad.bansod/videos/1800706399987528), before lowering their hindquarters in order to defecate. In all scats we found them to be clumped at one location (Fig 2A) and the elongated part, which exits the anus last, shows the location and direction faced by the defecating individual (Fig 2A). On the basis of the combined scratch marks on the ground and the scat orientation, the azimuth of the leopard’s body was determined (Fig 2B). During our field study, we located 49 scats with obvious scrape marks on the ground showing the direction of the head of the leopard and also studied the scat in order to confirm the directionality of the individual with the orientation of the scat (Fig 1).

We also noted the direction of movement of 31 different individual Leopards (only walking, Fig 1) when sighted randomly along the trails during our patrols. All encounters were random and no leopards were approached purposefully or disturbed.

### Statistical analysis

In order to test the effect of space orientation of the Leopards and the scats we used circular statistics implemented in circular library for R [26]. As a uniformity test we used rao spacing test with α = 0.01 significance level. We also used Watson Test [27, 28] to test the differences in the Leopards’ magnetic orientation during defecation and during movement. In order to check relationships between the leopard’s magnetic orientation and localisation (longitude and latitude) we used Mardia’s Rank Correlation Coefficient [27].

In order to avoid inconsistency resulting from sample size effect (n = 49 and n = 31), we performed a priori power test to determine the minimum sample size required to test our hypothesis. For this purpose we used G*Power 3.1.9.7 software [29]. As the effect size in the procedure we used Cohen’s d: Cohen’s d = (Mean_2_—Mean_1_) ⁄ SD_a_ where SD_a_ = √((SD_1_^2^ + SD_2_^2^) ⁄ 2). Furthermore we also used setting with α = 0.05 and nonsphericity correction ε = 1.

The result showed that the minimum samples size required to have an 95% chance of detecting differences was 32, which is below our data included for describing orientation during defecation (n = 49) and equal to the data showing walking direction (n = 31).

## Results

**Fig 3:**
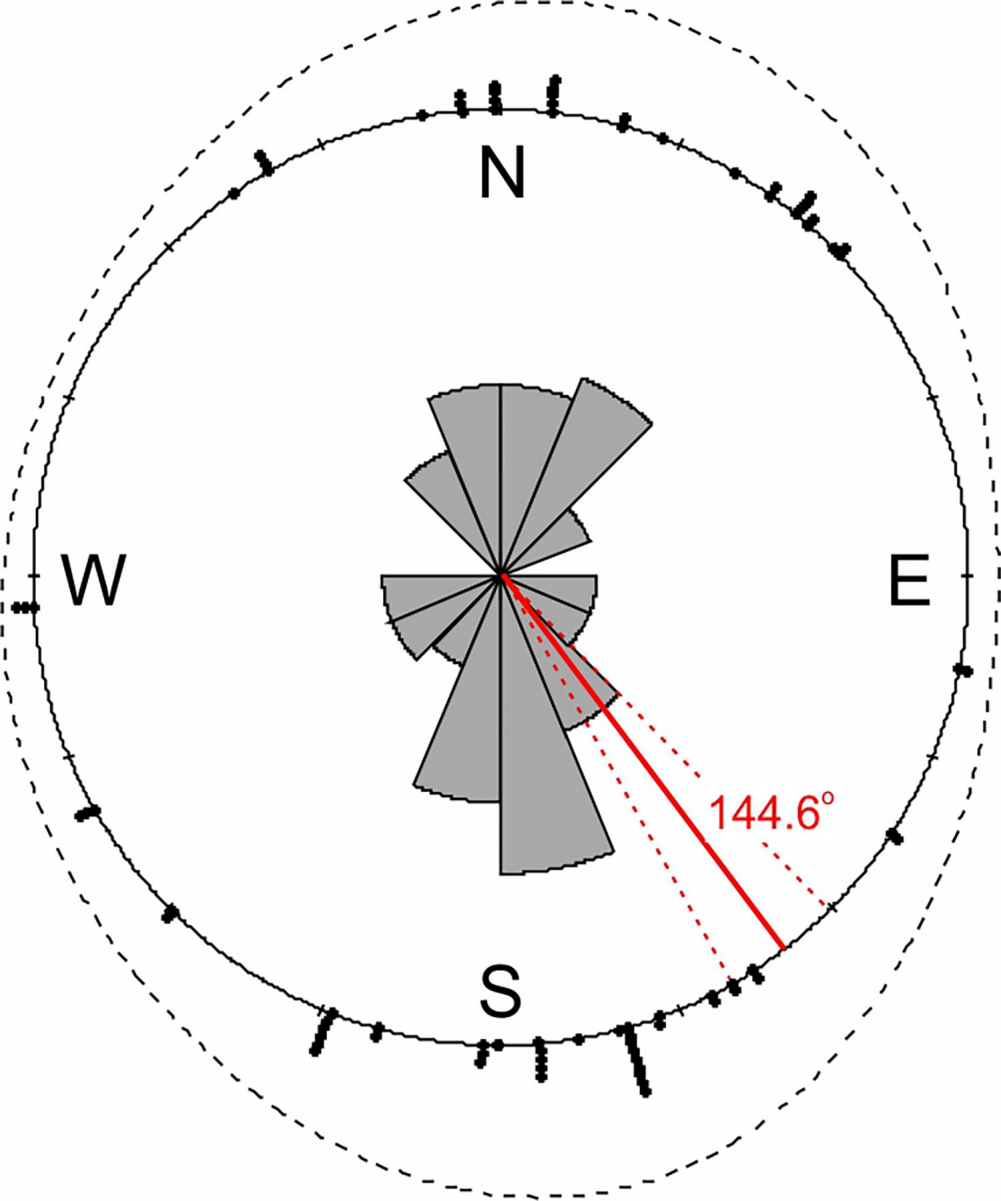
Distribution of Leopard (*Panthera pardus fusca*) orientation for all observations (walking and defecating). The solid red line denotes the circular mean, while the dashed red line represents the standard deviation; the black dashed line reflects the density of observations.

The circular mean (±SD) for leopards orientation was (144.6 ± 1.91^o^ azimuth) and according with Rao’s Spacing Test of Uniformity this orientation was significant (Rao = 237.5, p < 0.01, Fig 3).

**Fig 4:**
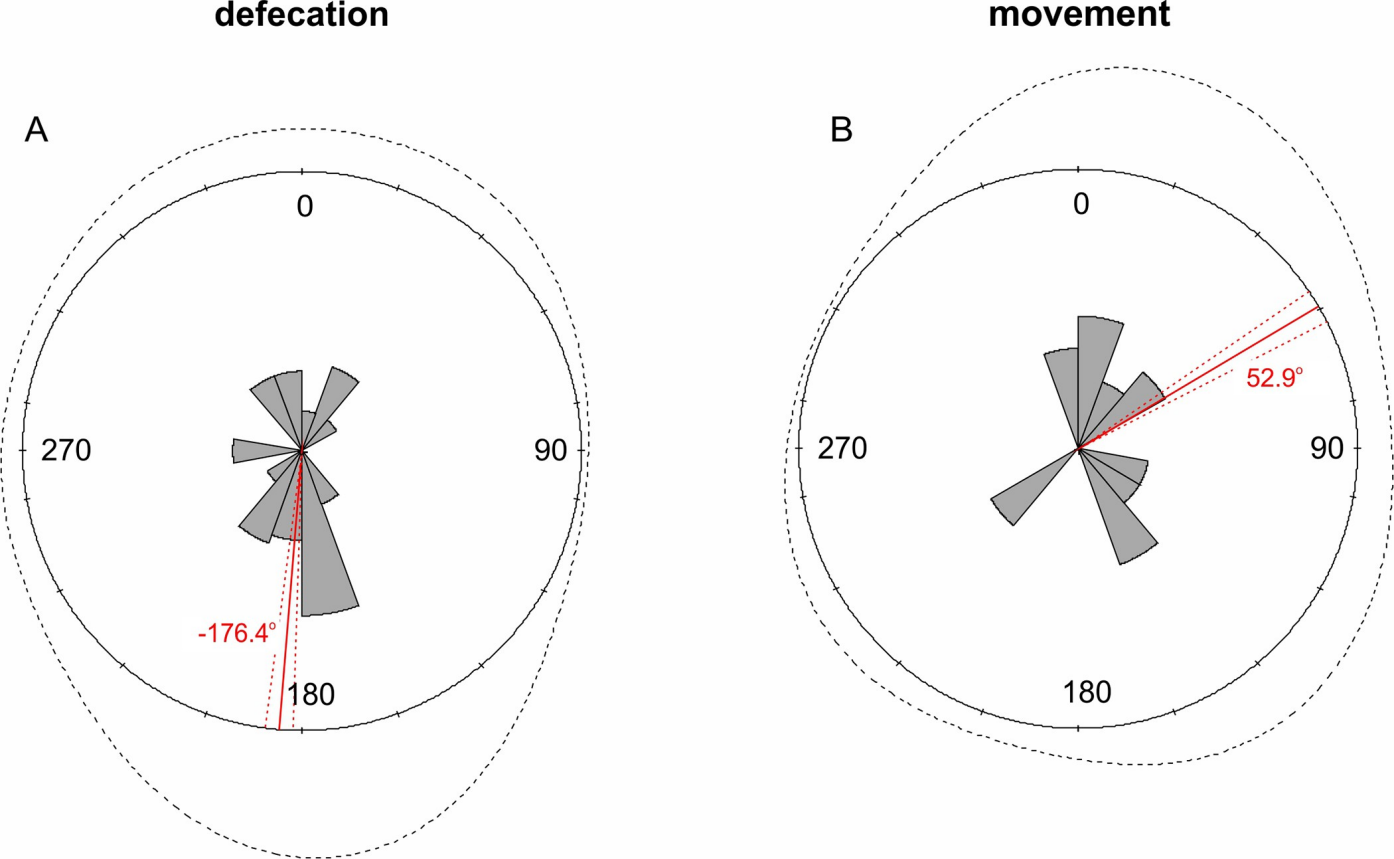
Differences between Leopard (*Panthera pardus fusca*) orientation (A) during defecation and (B) movement. The solid red line denotes the circular mean, while the dashed red line represents the standard deviation.

The circular mean (±SD) for leopards orientation during defecation was -176.4 (±1.46, N = 49) and was significant (Rao = 249.8, p < 0.01, Fig 4A); while circular mean (±SD) for leopards walking along trails was 52.9 (±1.47, N = 31) but was not significant (Rao = 168.3, p > 0.01, Fig 4B).

**Fig 5:**
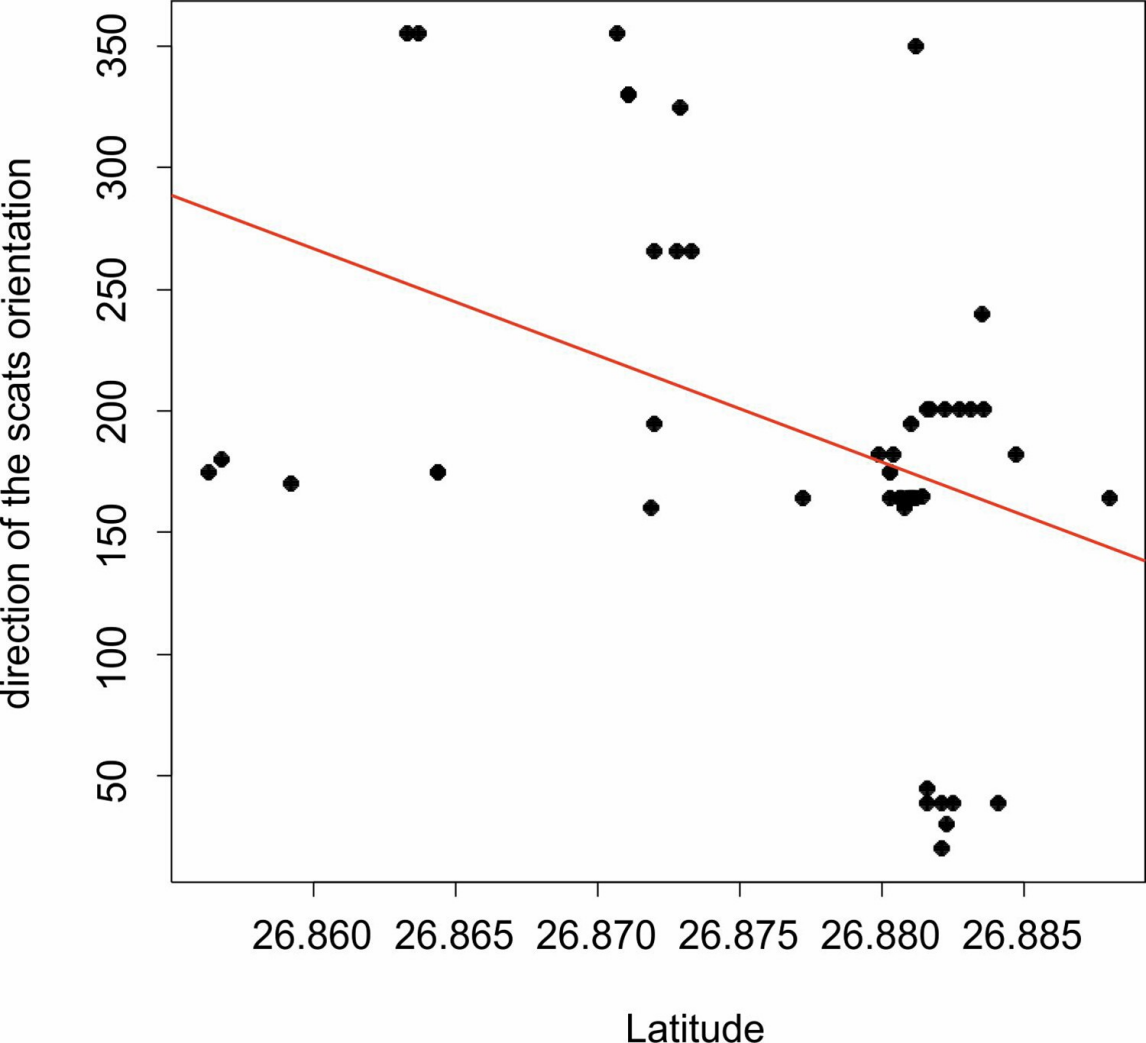
Effect of latitude on orientation of Leopards (*Panthera pardus fusca*) during defecation. The positioning of the body during defecation is related to the location of the leopard. Individuals observed more northwards, positioned their bodies more north-south axis than leopards further south.

Differences in orientation between defecation and movement was statistically significant (Watson Test = 0.68, p < 0.01). Orientation during defecation was associated with latitude of Leopard localization (r = -0.48, p-value for Mardia’s Rank Correlation Coefficient = 0.0001, Fig 5), while Longitude did not influence orientation during defecation (p-value for Mardia’s Rank Correlation Coefficient = 0.41).

## Discussion

Our results illustrated that the Indian leopard responds to GMF and that during defecation, oriented their bodies on the north-south linear axis, usually to the south (-167.5 i.e., 193^o^). There were several facing west but none to the east. During observed randomly walking along jeep trails the leopards did not show any directional preference. In other words, "defecation" is oriented to the south while "walking" was random and conforms to the contours of the trail on which it walked.

The directionality of defecation in the leopards was not exactly on the north-south axis but slightly deviated towards south-south east [30], having performed a meta-analyses of 23 vertebrate species whose magnetic alignment was reported in the scientific literature, found a significant clockwise shift from the north-south axis with a mean deviation of 9^o^. Although our data falls outside this average, it is close to it. The authors suggested that the shift could reflect an individual- or species-level bias resulting from lateralization of the GMF information in the central processing mechanism, which to date is poorly understood.

As previously mentioned, GMF studies on carnivores are rare, and on leopards this is the first study. Initially, most of the studies in dogs [31], foxes [32, 33] and coyotes (*Canis latrans*; [34]) reported of their homing capabilities with an inference to GMF. [8] found that In Red Foxes the hunting of rodents was non-randomly distributed with a significant preference from the northeast and they proposed the hypothesis that direction improved target-distance estimation mediated by a photoreceptor-based GMF system. We also checked whether the orientation of the scats depends on the geographic location of the leopard (latitude and longitude). It appears that latitude influenced scat orientation i.e., if the scats is localized more to the south, the orientation/azimuth of body orientation during defecation scats is also towards the south, and vice versa. It is additional proof that the leopards are sensitive to the GMF for basic physiological activities such as defecating. We also speculate that GMF is most probably also used for more important purposes such as delineating territorial boundaries [24], mapping the surroundings, for navigating and hunting in the neighbouring human settlements [25], and other survival and fitness related behaviours.

It should be also noted that the Rajasthan Forest Department have laid extensive infrastructure for CCTV and other monitoring devices in several locations within the boundaries of JFR. Thus, we suspected that the electromagnetic field generated by this infrastructure might interfere with the Leopard’s GMF perception, as is the case with influence of artificial magnets for hatchling sea turtles [35], rodents [36], and dogs [21]. However, studies on the influence of the electromagnetic field on various levels of biological organization from molecules to whole organisms have not shown that the electromagnetic field generated by the infrastructure television in any way disturbs the perception of other senses [37].

In conclusion, we show that the Indian leopard conforms in general to the findings of orienting the body in relation to the earth’s north-south geomagnetic field. Future studies should try and determine how the leopards create a map based on the GMF, and whether it can be influenced by external, artificially generated, electromagnetic forces. Also, of importance is to try and elucidate the differences in the mechanisms that reflect a bias in the alignment mechanism and how it is possibly influenced by laterality.

## Supporting information

S1 Table Detailed information about all samples used in the study. (XLS)
